# Periodontal changes after surgically assisted rapid maxillary expansion (SARME)

**DOI:** 10.1007/s10006-015-0506-5

**Published:** 2015-05-23

**Authors:** Thomas Jensen, Lars Hjelm Johannesen, Maria Rodrigo-Domingo

**Affiliations:** Department of Oral and Maxillofacial Surgery, Aalborg University Hospital, Hobrovej 18-22, 9000 Aalborg, Denmark; Department of Oral and Maxillofacial Surgery, Department of Dentistry, Aarhus University, Aarhus, Denmark; Department of Clinical Medicine, Aalborg University, Aalborg, Denmark

**Keywords:** Maxillary expansion, Malocclusion, Orthognathic surgery, Relapse

## Abstract

**Purpose:**

The objective of the present study was to evaluate the health status of the periodontal tissue after surgically assisted rapid maxillary expansion (SARME).

**Methods:**

Periodontal status was assessed after an average of 25 months (range, 6–66) in 61 patients who underwent SARME by plaque index, gingival index, probing depth, and probing attachment level. In the maxilla, six measurements were made at the central incisor, second premolar, and first molar. Corresponding measurements were made in the mandible as control. The measurements were estimated and expressed with 95 % confidence interval (CI). Additionally, maxillary occlusal radiographs of the maxillary central incisors were evaluated for signs of root resorption.

**Results:**

There was no statistically significant difference between experimental and control teeth with respect to plaque index, probing depth, or attachment level. The gingival index of the maxillary central incisor was significantly higher compared to control (CI 0.175 (0.09–0.26), *p* value *p* < 0.001). External apical root resorption of the anterior maxillary teeth was observed in 36 % of the patients.

**Conclusions:**

Within the limitations of a retrospective study, the present study seems to demonstrate that SARME does not affect the health status of the periodontal tissues. However, further randomized long-term studies are needed before final conclusions can be provided.

## Introduction

Transverse maxillary hypoplasia, in adolescents and adults, is characterized by a narrow maxillary arch, unilateral or bilateral cross-bite, a high narrow palatal vault, and crowded misaligned teeth. Multisegmental Le Fort I osteotomy is the most frequently used surgical method for correction of transverse discrepancies in adults. However, extensive transverse expansion of the maxilla with a multisegmental Le Fort I osteotomy is often associated with postsurgical instability and relapse [1–3]. Surgically assisted rapid maxillary expansion (SARME) has therefore become a common surgical method in treating patients with transverse maxillary hypoplasia with closed midpalatal suture [4, 5]. SARME is a combination of orthodontics and distraction osteogenesis which provides dental arch space for alignment of the teeth and minimizes transverse maxillary relapse in skeletally mature patients.

Nonsurgical rapid maxillary expansion, by orthodontic opening the maxillary midline suture, in skeletally mature patients may cause periodontal ligament compression, buccal root resorption, fenestration of the buccal cortex, and extrusion of the teeth due to the increased skeletal resistance [4–6]. These complications can be minimized or avoided by surgically releasing the osseous structures that resist the expansion force. The effects of mechanical forces on the anchoraging teeth and the periodontal tissues during SARME have previously been evaluated, demonstrating various periodontal complications including gingival recession, periodontal bone defects, and root resorption of the maxillary central incisors [7–11]. Furthermore, loss of tooth vitality, discoloration, gingival recession, and external root resorption have also been reported as a consequence of the medial osteotomy between the maxillary central incisors [4, 8, 12]. It has been claimed that bone-borne distraction devices may reduce the risk of the periodontal complications since the force is not applied directly on the teeth, and a slower distraction rate could be rational due to the proximity of the median osteotomy to the periodontal ligament [8, 9, 13, 14]. However, a previously published systematic review shows that there is no strict consensus for the surgical protocol, the distraction rate, and the consolidation of the SARME [5].

In the previously published studies, the health status of the periodontal tissue has never been compared to periodontal findings in the remaining dentition. Therefore, the objective of the present study was to test the hypotheses that there is no difference in the periodontal health status between the maxillary central incisors and teeth fixated to the tooth-borne distraction device compared to the corresponding teeth in the lower jaw after SARME.

## Methods

### Patients

Seventy-three consecutive skeletally mature nonsyndromic patients (28 males and 45 females) with a maxillary transverse deficiency of more than 5 mm underwent SARME at the Department of Oral and Maxillofacial Surgery, Aalborg University Hospital, Denmark. The average age of the patients at the time of surgery was 23 years (range 14–53). Sixty-one patients responded to a follow-up call at least 6 months after SARME, with an average period of 23 months (range 6–66). All clinical and radiographic evaluations were performed independently by one of the two authors (TJ, LJ).

### Surgical technique

**Fig. 1:**
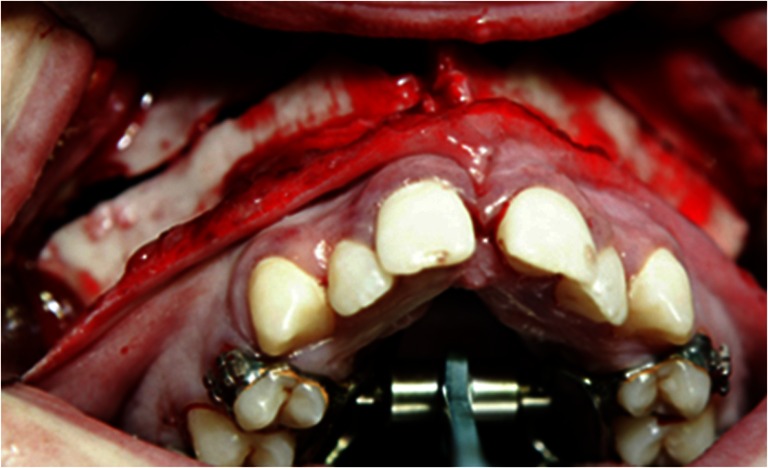
The final osteotomy. The distraction device was activated until a 1-mm midline diastema was achieved

**Fig. 2:**
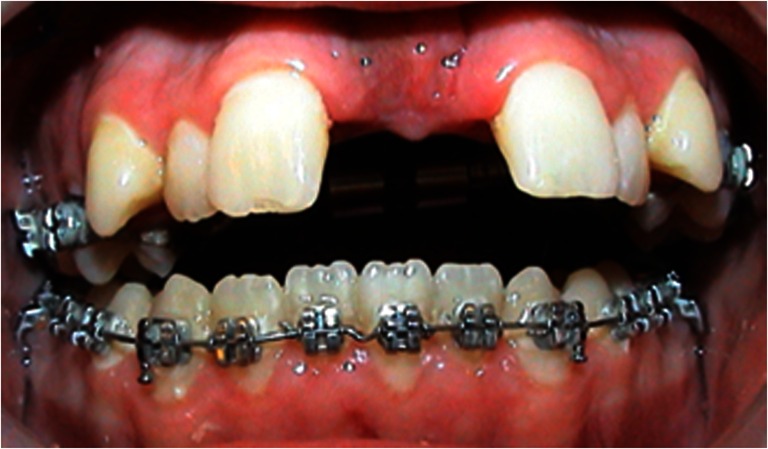
Final transverse expansion at the end of the distraction phase

All patients underwent SARME under general anesthesia with nasotracheal intubation. The surgical procedure was performed by different surgeons using a similar surgical technique. A tooth-borne Hyrax palatal expansion device was cemented by an orthodontist prior to surgery. A vestibular incision was made from the right first molar to the left first molar. The mucoperiosteum was reflected exposing the maxilla and anterior nasal floor. A horizontal osteotomy was made at Le Fort I level, 5 mm above the root apices, and parallel to the maxillary occlusal plane. The osteotomy was completed with a chisel, and the pterygoid plates were separated from the tuberosity with a curved osteotome. The nasal septum was released with a nasal osteotome before the maxilla was mobilized to prevent any bony interference. The distraction device was activated to induce pressure on the midpalatinal suture, before a midpalatal osteotomy was made through the outer cortex, initially with bur, and finally with a chisel until the segments could be moved without any bony resistance. Interfering bone was removed from the zygomatic buttress to allow expansion, and the distraction device was activated until a 1-mm midline diastema was achieved (Fig. 1). After a latent period of 5–7 days, the patients were instructed to activate the distraction devices by 0.5-mm increments twice a day until the planned expansion was achieved (Fig. 2). The segments were retained by the distraction device for approximately 6 months. Further active orthodontic treatment was initiated after 6 months to finalize the occlusion or to prepare the patient for a second-stage osteotomy.

### Clinical evaluation

The clinical examination of the periodontal health status at the follow-up call included plaque index, gingival index, probing pocket depth, and probing attachment level according to the system described by Löe [15]. These registrations were measured to the nearest millimeter, mesio-buccally, mid-buccally, disto-buccally, mesio-lingually, mid-lingually, and disto-lingually at the maxillary central incisor, second premolar, and first molar. Corresponding measurements were made in the mandible as control.

### Radiographic evaluation

The radiographic examination included maxillary occlusal radiographs for evaluation of external apical root resorption (EARR). The radiographs taken at the follow-up visit were compared with the radiographs obtained at the first postoperative day. Maxillary incisors that showed EARR were divided into three groups according to the classification described by Sharpe [16]: group 1—slight blunting of the root apex, group 2—moderate resorption up to one third of the root length, and group 3—extreme resorption beyond one third of the root length.

### Statistical analysis

A score per tooth and patient was constructed based on the six registrations in the Löe and Silness system available for each tooth (mesio-buccally, mid-buccally, disto-buccally, mesio-lingually, mid-lingually, and disto-lingually). For the plague and gingival indices, each with levels 0–3, the mean of the six measurements was taken as score. For attachment level and pocket depth, the possible values are 0–3, 4–5, and >5 mm. A weighted mean of the six registrations was computed with weights 2, 4.5, and 7 for the respective levels. The scores can therefore range between 2 (all registrations in the first level) and 7 (all registrations in the last level). For example, a tooth with values (0–3, 0–3, 0–3, 4–5, 0–3, 0–3 mm) will have a score of 2.42. We then computed the differences in scores between the maxilla and the mandible for the six teeth (central incisors, second premolar, and first molar, for the right and left sides). A mixed-effects model with differences in score as outcome, tooth as fixed effect, and patient, and side as random effects, was used. The statistical analyses were done in STATA version 13.1. The mean difference in score between the maxilla and mandible per tooth was estimated and expressed with 95 % confidence interval (CI) and *P* value. *P* values below 0.05 were considered statistically significant.

## Results

**Fig. 3:**
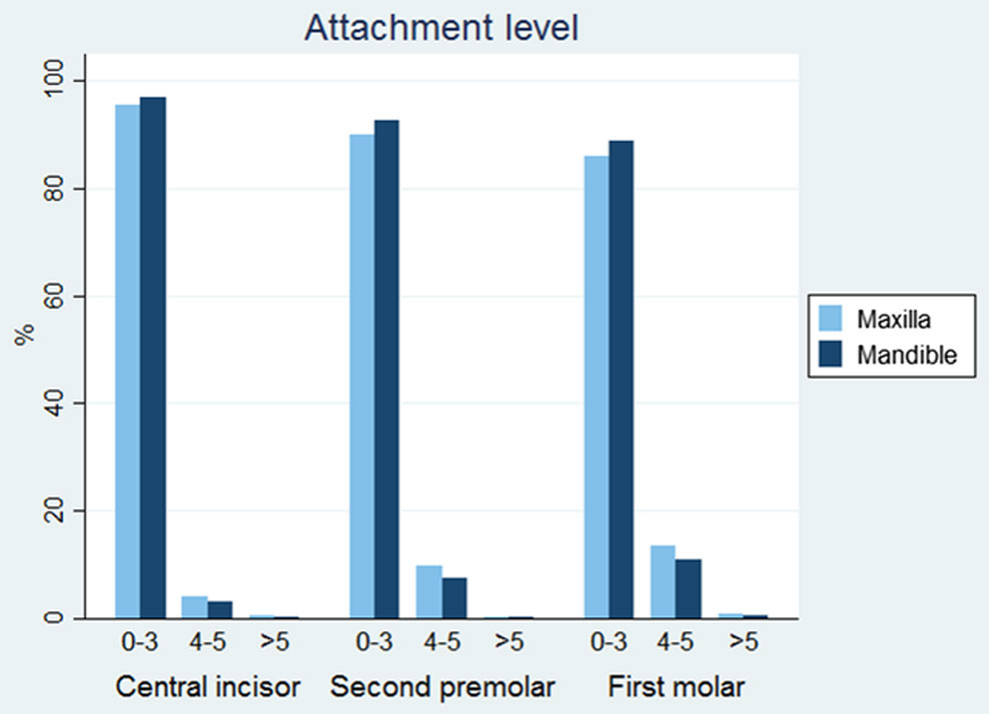
Attachment level

**Fig. 4:**
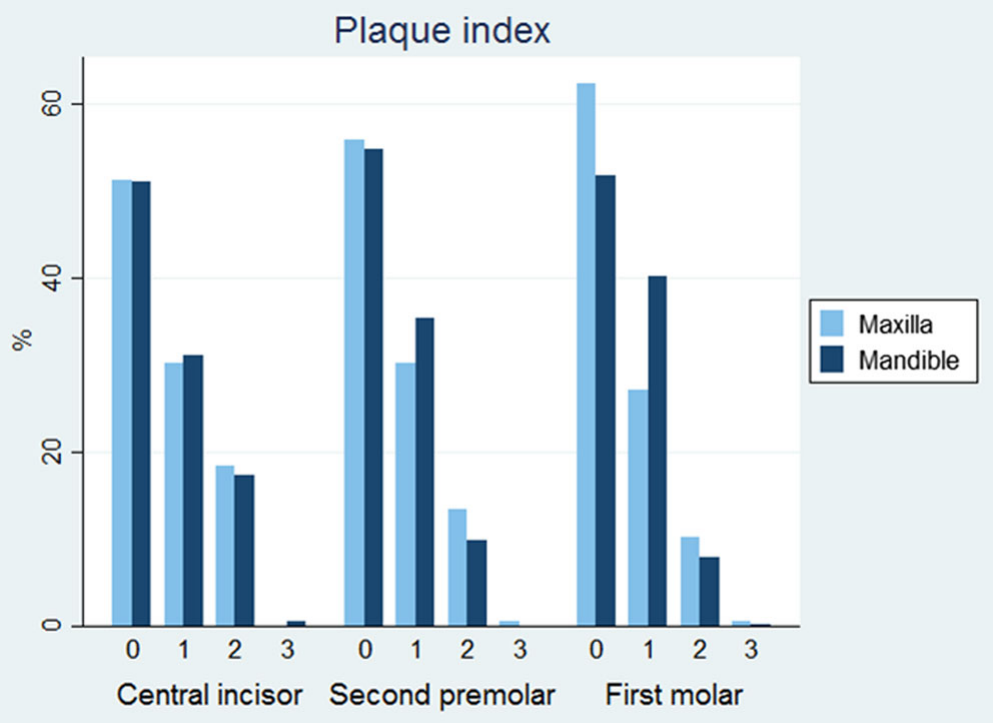
Plaque index

**Fig. 5:**
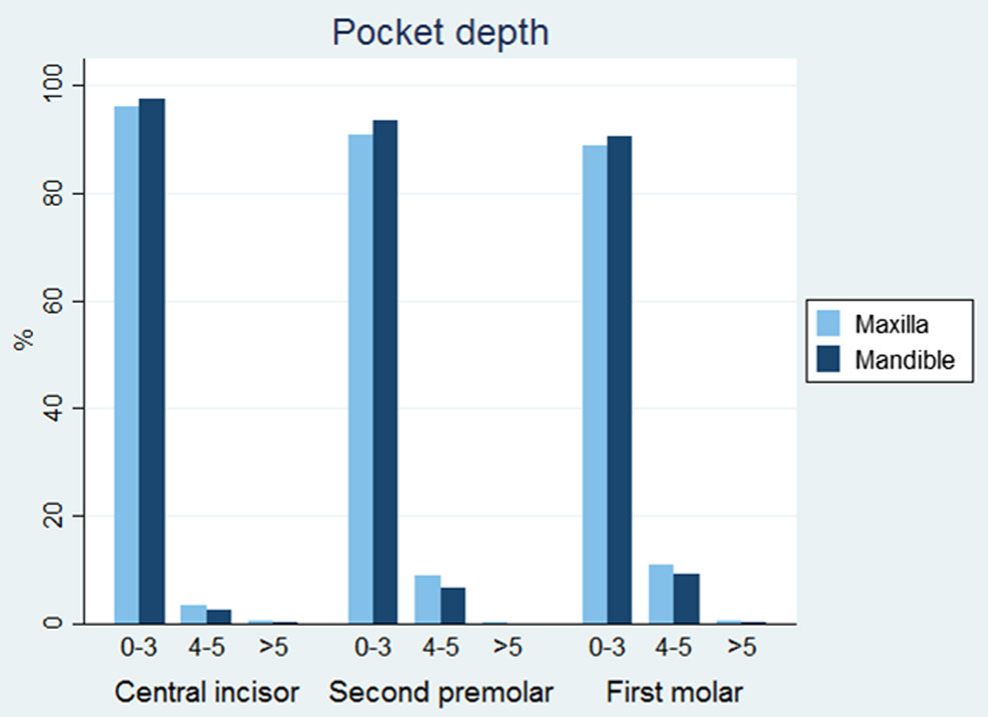
Pocket depth

**Fig. 6:**
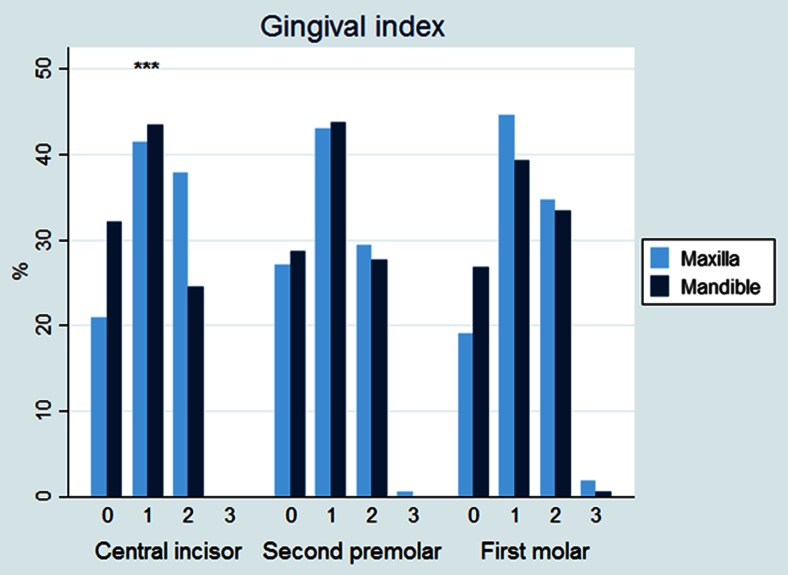
Gingival index

**Fig. 7:**
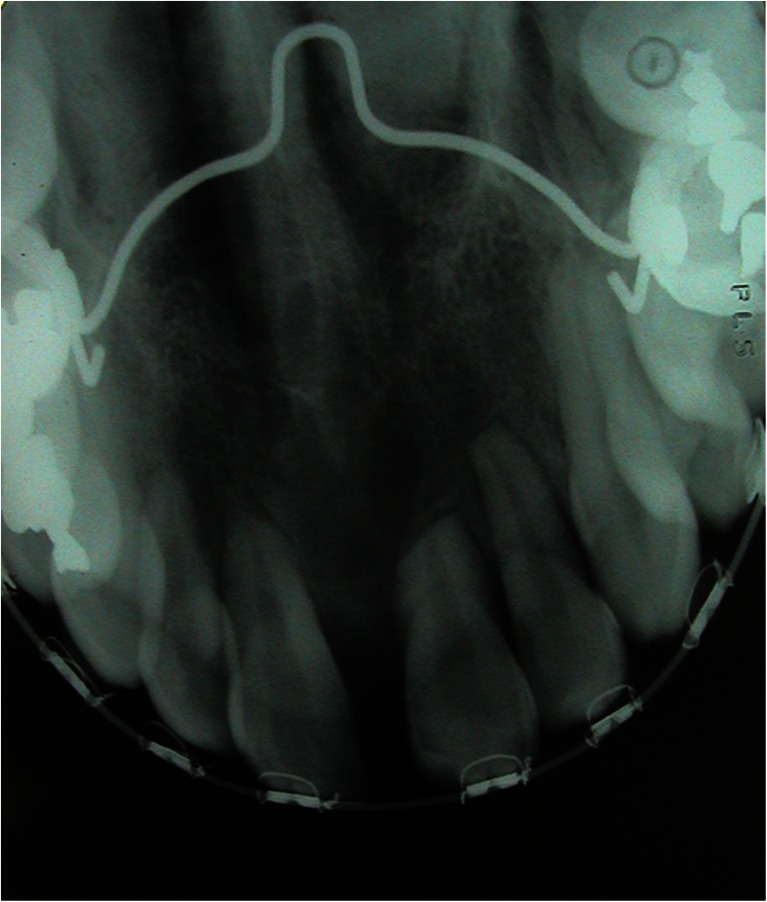
Extreme resorption beyond one third of the root length

The results are outlined in Table 1. There was no statistically significant difference between experimental and control teeth with respect to plaque index, probing pocket depth, or probing attachment level (Figs. 3, 4, and 5). The gingival index of the maxillary central incisor was significantly higher than that of the mandible (CI 0.175 (0.09–0.26), *p* < 0.001) (Fig. 6). EARR of the anterior maxillary teeth was seen in 36 % of the patients (Table 2). Extreme resorption beyond one third of the root length was seen in two cases (Fig. 7).

**Table 1:** The mean difference in score between maxilla and mandible per tooth

Probing attachment level	Mean	*P* value	95 % CI
Central incisor	0.044	0.313	(−0.042–0.131)
Second premolar	0.068	0.121	(−0.018–0.155)
First molar	0.078	0.074	(−0.008–0.165)
Probing pocket depth			
Central incisor	0.044	0.263	(−0.033–0.122)
Second premolar	0.072	0.070	(−0.006–0.149)
First molar	0.051	0.196	(−0.026–0.129)
Plaque index			
Central incisor	−0.001	0.974	(−0.085–0.082)
Second premolar	0.036	0.405	(−0.048–0.119)
First molar	−0.079	0.063	(−0.163–0.004)
Gingival index			
Central incisor	0.175	0.000	(0.094–0.256)^a^
Second premolar	0.046	0.260	(−0.034–0.127)
First molar	0.079	0.055	(−0.002–0.16)

^a^The gingival index of the maxillary central incisor was significantly higher

**Table 2:** Radiologic evaluation of EARR

EARR type	0	1	2	3	Total
Number	78	30	12	2	122

According to the classification by Sharpe [16]

## Discussion

The present study demonstrates that the health status of the periodontal tissue was not significantly affected by SARME, although the gingival index of the maxillary central incisor was significantly higher compared to the mandible. Apical root resorption of the anterior maxillary teeth was frequently observed. However, in most of the cases, it was only a slight resorption of the apex as often seen after conventional orthodontic treatment.

Malocclusion and crowed misaligned teeth have been shown to compromise sufficient oral hygiene due to difficulties of plague removal. Dental alignment facilitates plaque removal and may promote to a healthier periodontium. Insertion of orthodontic appliances, as well as the mechanical orthodontic force applied to the teeth, can make it difficult to preserve good oral hygiene and contribute to chronic infection, irreversible loss of attachment, and gingival recession [17]. Rapid maxillary expansion, by orthodontic opening of the maxillary midline suture, in skeletally mature patients has shown to cause periodontal ligament compression, buccal root resorption, fenestration of the buccal cortex, and extrusion of teeth due to the increased skeletal resistance [4–6]. The combination of poor oral hygiene and SARME will therefore pose a significant risk of periodontal damage.

Assessment of the periodontal health status after SARME has previously been reported in a few studies using different distraction devices, surgical techniques, and distraction rate protocols [7–11]. Periodontal complications after SARME mainly involved the mesial aspect of the central incisors, and gingival recession seems to be the most common periodontal complication [7–11]. In the present study, the gingival recession was not assessed; however, measurement of pocket depth and probing attachment level after SARME demonstrated no significant differences between the maxillary central incisors and teeth fixated to the distraction devices compared with corresponding teeth in the lower jaw. In the majority of the previously published studies, the frequency of gingival recession after SARME seems to be very low [8–11]. However, gingival recession of 29 % has been presented in one study, where 35 patients with an average age of 24 years underwent SARME with solely a midpalatal osteotomy [7]. The distraction device was activated during surgery until the planned transverse expansion was obtained and locked in this position for 4 months. Dental study casts and intraoral photographs were used to measure the length of the clinical crown, demonstrating gingival recessions in the upper dental arch of 29 % [7]. In this study, only the midpalatal suture of the osseous structures that resist the expansion was surgically released, and slow expansion of the maxilla by the use of distraction osteogenesis was not utilized [7]. Hence, the results of this study seem not to be reliable for the effect of the mechanical orthodontic force on the periodontal tissue of the supporting teeth during SARME.

Asymmetric separation of the midpalatal suture during distraction may induce pressure on the mesial gingival attachment of the maxillary central incisors and increase the risk of periodontal complications [18]. Maintaining the integrity of the alveolar bone and the periodontal ligament on the roots of the central maxillary incisors during splitting of the midpalatal suture has been emphasized as an important factor to reduce periodontal breakdown [8]. Moreover, orthodontic root separation before SARME and midpalatal osteotomy has been recommended in cases with close root proximity of the central incisors [18]. Bone loss and periodontal complications due to eccentric midpalatal osteotomy during SARME have previously been described in the literature [8, 9]. Verlinden et al. found considerable periodontal bone loss between the central incisors due to an eccentric midpalatinal osteotomy in two cases, causing separation of the bone from the root surface and later removal of the central incisors [9]. Ramieri et al. found a lack of alveolar bone regeneration along the exposed roots of the central incisors in four patients with eccentric interdental fractures [8]. In the present study, we used a surgical method, where the distraction device was activated to induce maximal pressure on the midpalatinal suture, before an osteotomy was made with a bur through the outer cortex and finally with chisel along the midpalatal suture. Thus, enabling control of the midpalatal osteotomy and no eccentric separation of the midpalatal suture occurred in our study.

It has been hypothesized that bone-borne distraction devices may reduce the risk of periodontal complications, since the force is not applied directly on the teeth [8, 13, 14]. Studies assessing the periodontal effects of SARME have been used either tooth-borne [7, 11], bone-borne [8, 9], or both [10], but no studies have compared the periodontal health status after SARME with either tooth- or bone-borne distraction devices. In the present study, we used a tooth-borne distraction device and the periodontal effect of SARME seems to be comparable to what has been reported previously on this topic using either tooth- or bone-borne distraction devices [8–11]. Moreover, a systematic review has concluded that there is only weak evidence for less buccal tipping of the teeth used as anchor teeth in tooth-borne expansion compared to bone-borne expansion [5] and that short-term skeletal and dental changes following the two treatment modalities were comparable [19].

A slower distraction rate of 0.33 mm/day has been advocated due to the proximity of the median osteotomy to the periodontal ligament [9]. Distraction rate protocols vary among the published studies, and no strict consensus exists for the surgical protocol, the distraction protocol, and the consolidation of the SARME [5].

In the present study, the gingival index of the maxillary central incisor was significantly higher compared to the mandible. Although this difference seems not to influence the other periodontal measurements and the present health status of the periodontal tissue, it can be due to reduced oral hygiene or a vulnerable mucosa due to stretching during distraction.

EARR of the anterior maxillary teeth was seen in 36 % of the patients in our study. However, extreme resorption beyond one third of the root length was only seen in two patients. Similar results have been described by Verlinden et al. demonstrating 29 % EARR after SARME [9]. Maxillary central and lateral incisors seem to be more susceptible to EARR, and the radiographic signs of EARR seem to be similar to what can be seen after conventional orthodontic treatment.

SARME is a surgical procedure with low morbidity especially when compared with other orthognathic procedures. However, hemorrhage, extrusion of teeth, unilateral expansion, and orbital compartment syndrome have previously been reported with SARME [10, 20]. Most of the complications in the present study have previously been described (Table 3). Severe epistaxis was seen on the ninth postoperative day, while the patient was activating the distraction device. The bleeding did not stop before a compressive balloon was place.

**Table 3:** Complications after SARME

Type of complications	No. of cases
Postoperative hemorrhage	1
Severe bleeding on the ninth postoperatively day	1
Sinusitis	2
Extrusion of teeth fixed to the distraction device	1
Inability to open the midpalatal suture	1
Massive hematoma	2
Loosening of distraction device	3
Buccal emphysema	2
Palatal mucosal ulceration due to pressure from distraction device	1

## Conclusion

In conclusion, SARME seems not to affect the health status of the periodontal tissue, but EARR of the anterior maxillary teeth was frequently observed, although in most of the cases, it was only a slight resorption of the apex as often seen after conventional orthodontic treatment. However, further randomized long-term studies are needed before final conclusions can be provided.
